# CO_2_ and H_2_O: Understanding Different Stakeholder Perspectives on the Use of Carbon Credits to Finance Household Water Treatment Projects

**DOI:** 10.1371/journal.pone.0122894

**Published:** 2015-04-30

**Authors:** Sarah K. Summers, Rochelle Rainey, Maneet Kaur, Jay P. Graham

**Affiliations:** 1 Department of Global Health, Milken Institute School of Public Health, The George Washington University, Washington, D.C., United States of America; 2 United States Agency for International Development (USAID), Washington D.C., United States of America; 3 Berkeley Air Monitoring Group, Berkeley, California, United States of America; University of Waterloo, CANADA

## Abstract

**Background:**

Carbon credits are an increasingly prevalent market-based mechanism used to subsidize household water treatment technologies (HWT). This involves generating credits through the reduction of carbon emissions from boiling water by providing a technology that reduces greenhouse gas emissions linked to climate change. Proponents claim this process delivers health and environmental benefits by providing clean drinking water and reducing greenhouse gases. Selling carbon credits associated with HWT projects requires rigorous monitoring to ensure households are using the HWT and achieving the desired benefits of the device. Critics have suggested that the technologies provide neither the benefits of clean water nor reduced emissions. This study explores the perspectives of carbon credit and water, sanitation and hygiene (WASH) experts on HWT carbon credit projects.

**Methods:**

Thirteen semi-structured, in-depth interviews were conducted with key informants from the WASH and carbon credit development sectors. The interviews explored perceptions of the two groups with respect to the procedures applied in the Gold Standard methodology for trading Voluntary Emission Reduction (VER) credits.

**Results:**

Agreement among the WASH and carbon credit experts existed for the concept of suppressed demand and parameters in the baseline water boiling test. Key differences, however, existed. WASH experts’ responses highlighted a focus on objectively verifiable data for monitoring carbon projects while carbon credit experts called for contextualizing observed data with the need for flexibility and balancing financial viability with quality assurance.

**Conclusions:**

Carbon credit projects have the potential to become an important financing mechanism for clean energy in low- and middle-income countries. Based on this research we recommend that more effort be placed on building consensus on the underlying assumptions for obtaining carbon credits from HWT projects, as well as the approved methods for monitoring correct and consistent use of the HWT technologies in order to support public health impacts.

## Introduction

Carbon credits, or offsets, are a market-based method for reducing the amount of greenhouse gas (GHG) emissions into the atmosphere. A carbon credit represents the removal of one ton of carbon dioxide or its equivalent from the atmosphere. Entities desiring to purchase carbon credits have a variety of motivations, including: 1) countries aiming to comply with regulations or international obligations [1]; 2) companies wishing to offset their own emissions from production and/or distribution to raise their environmental and social profile; and 3) individuals desiring to offset emissions associated with daily living. With the adoption of the Doha Amendment to the Kyoto Protocol, which commits countries to further emissions reductions through 2020 [2], it is appropriate to direct renewed attention towards the evaluation of carbon credits as a development and environmental conservation mechanism.

While the compliance market for carbon credits was created as a regulatory mechanism of the Kyoto Protocol, a smaller, voluntary market emerged for governments, businesses, NGOs and individuals seeking to reduce their carbon footprint by offsetting their emissions. In response to this new unregulated voluntary market, standards and organizations “verifying” the production of carbon credits began to proliferate. Verification of carbon credits implies that the entity or organization issuing the carbon credits has verified that the claimed emissions reductions and any additional social benefits have been achieved. Examples of these standards and markets are Verified Carbon Standard, VER+, Chicago Climate Exchange and Gold Standard [3].

Globally, around 3 billion people cook and heat their homes using open fires and inefficient stoves burning low quality fuels like biomass (wood, animal dung and crop waste) and coal [4]. These energy sources generate air pollutants that contribute to climate change and negatively impact health. Many of these people also boil their drinking water to make it safe to consume, so organizations involved in the carbon market explored alternatives that provide safe drinking water while generating fewer greenhouse gas emissions, creating carbon credits that could be sold and traded.

The largest household water treatment (HWT) projects registered for carbon credits are utilizing the Gold Standard [5]. The Gold Standard Foundation is a non-profit organization registered in Switzerland that certifies emissions reductions for the international compliance and voluntary carbon markets. The Gold Standard Foundation first issued guidelines with specific applications and reference to safe drinking water projects in the 2010 version of their methodology for improved cook stoves [6]. The guidelines and practices established by Gold Standard methodology are generally considered the most rigorous in terms of monitoring and verifying emissions reductions [3]. Some examples of the more rigorous standards include higher standards for verifying the microbiologic safety of water and taking life cycle emissions of the project into account. [6–9] The methodology for safe drinking water projects was revised in November of 2011 [7] and updated guidelines for conducting usage surveys and monitoring water quality were issued in 2014 [8,9].

Critics of carbon credits for household water treatment point to a lack of transparency in the processes for validating and verifying emissions reductions. This criticism is most pronounced with respect to the monitoring and evaluation procedures established by carbon credit standards [10–13]. This study explores the perspectives of carbon credit and water, sanitation and hygiene (WASH) experts related to monitoring and evaluation methods for HWT carbon credit projects as described in the Gold Standard methodology. This paper focuses only on monitoring and evaluation procedures in Gold Standard methodology. The results and conclusions are not intended to be generalized to all voluntary standards nor to the Clean Development Mechanism policies and procedures.

### Household water treatment and carbon emissions

There are a variety of technologies for improving the microbiological quality of drinking water at the point of use. These include boiling, chemical disinfection with chlorine, filtration, solar disinfection, or a combination of these methods. Boiling is by far the most common method used in resource-poor settings for treating drinking water. It does not require a new supply chain or repeated purchase and use of a product; however, in most cases it does require additional fuel beyond what is already used for cooking. The low-quality fuels used in these settings result in carbon emissions as well as particulate air pollution, with devastating health impacts [4].

**Fig 1:**
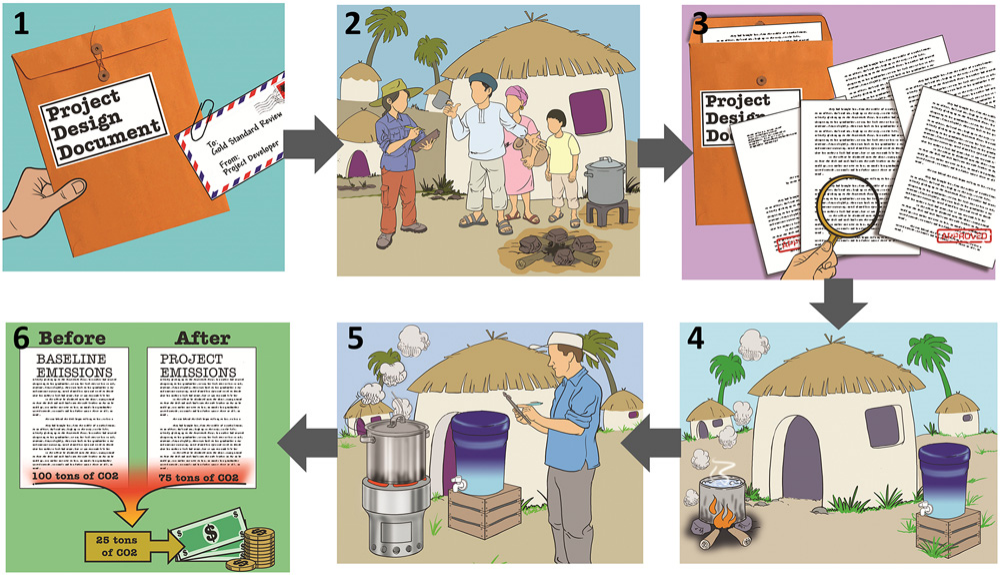
Description of steps for projects to obtain carbon credits for a household water treatment project. (A) Step 1: The project has to submit a Project Design Document (PDD) to Gold Standard for review (B) Step 2: Within the PDD the project developer must provide detail on the project location and baseline characteristics of end-users of the HWT. The characteristics of technology users include the baseline technology in use (type of stove/fuel) and user practices (time spent cooking/boiling water). (C) Step 3: A third party entity conducts stakeholder interviews and confirms that if the project were to move forward as proposed planned emissions reductions would be achieved. (D) Step 4: The project technology is installed and ready for use. (E) Step 5: A third party designated operational entity (DOE) periodically collects monitoring data on indicators of fuel and filter user throughout the stated life of the project. These indicators are used to calculate project level emissions. (F) Step 6: The project level emissions are subtracted from the baseline emissions and carbon credits are issued based on the difference.

Several carbon credit projects exist to reduce carbon emissions associated with boiling water, which they attempt to achieve through the dissemination of a technology, such as a water filter, that displaces boiling and reduces greenhouse gas emissions (Table 1). Vestergaard Frandsen, a manufacturer of a range of health products, proposes to reach one million households and approximately four million people with its LifeStraw Family filter in Kenya [14–15]. There are several steps, described in Fig 1, which HWT projects go through in order to earn carbon credits under the Gold Standard.

**Table 1:** Household water treatment projects applying for carbon credits using Gold Standard.

Project Developer	Technology	Country	Estimated Emissions Reductions^2^ Per Year	Status of Credits
co2balance	boreholes	Bangladesh; Malawi; Mozambique; Sierra Leone; Uganda	60,000^3^	Listed / Registered
Envirofit International	Lifestraw Family 1.0	Tanzania	92,933	Issued
Hindustan Unilever Limited	Pureit Water Purifier	Kenya	26,686,332	Listed
Hydrologic Social Enterprise	Ceramic filter	Cambodia	89,474	Issued
Impact Carbon	Not available	Indonesia; Sudan; Uganda	154,577;10,00;5,000	Listed
Paradigm Project	Ceramic filters, POU chemicals, community level (borehole, chemical)	Kenya	484,746	Issued
Paradigm Project	Not available	Guatemala	3,194,906	Registered
TerraClear	Ceramic filter	Lao PDR	33,541	Validated
Triple Quest	Biosand filter	Ethiopia; Ghana; Honduras; Kenya	10,000;9,740; 9,023; 7,583	Listed / Registered
Swiss Carbon Assets	Gravity driven ultrafiltration membrane	Uganda	6,254	Validated
Swiss Carbon Assets	chlorine dispensers	Uganda	51,415	Listed
Vestergaard Frandsen	Lifestraw Family 1.0	Kenya	2,073,328	Issued
Viability Africa	Biosand filter, sand+membrane filters	Kenya	120,939	Registered

Includes projects that have made documents publically available in the Markit Environmental Registry.^1, 4^

^1^ This information was current as of February 2015 and only reflects the available information on the Markit Environmental Registry. The authors recognize this information may have been updated or changed since the time of publication.

^2^ Emissions reductions are estimated based on all project technologies, which may include technologies other than water treatment devices.

^3^ This project includes six projects with an estimated 10,000 emissions reduction per project per year.

^4^ Project statuses in the Markit Environmental Registry are categorized as “Listed”, “Validated”, “Registered”, or “Issued”. A “Listed” project is in its earliest stages as a Gold Standard (GS) applicant after submission of a Local Stakeholder Consultation Report and completion of a GS pre-feasibility assessment. A project becomes “Validated” after a series of stakeholder consultations and feedback as well as an audit from an independent UN-accredit auditor, called a Designated Operational Entity (DOE). After a final document review by the GS, the project is then “Registered”. Finally, after another DOE audit and a GS review to verify project emissions reductions, the project is “Issued” CO_2_ credits.

A critical question in calculating emissions reductions is the current prevalence of boiling drinking water. A 2010 study estimating the scope of HWT practices found boiling water most prevalent in the Western Pacific region and least prevalent in Africa [16]. Data from 22 African countries were included in this sample; of these 22 countries only four had boiling rates over 10%: Malawi (10.2%), Uganda (39.8%), Zambia (15.2%), and Zimbabwe (10.4%)[16]. Of the current HWT projects registered under Gold Standard (Table 1) only three host countries have a boiling prevalence over 50%: Cambodia, Lao PDR, and Indonesia [16].

In carbon credit methodologies, the baseline water-boiling test (BWBT) is designed to measure “the quantity of fuel required to boil one liter of water for 10 minutes using technologies and fuels representative of the baseline scenario” [7]. The guidelines for the BWBT do not specify whether the 10 minutes is inclusive of the time it takes for the water to reach a boil nor the evidence base for establishing 10 minutes as the appropriate baseline.

The current literature assessing boiling practices in the field reflects similar ambiguities regarding definitions and time spent boiling. A 2007 study of 50 households in Vietnam found the mean self-reported time for boiling was 15.8 minutes [17]. A study of households in Zambia found that on average people spent 13 minutes per liter boiling water [18]. It is unclear, however, if this time was inclusive of the time it took water to reach the boiling point, or if it was the actual time water was boiling. A 2012 study in Cambodia reported an average boiling time of 20 minutes, but the range was 550 minutes [19]. The authors indicate this time is exclusive of time spent preparing to boil water (gathering or purchasing fuel) or waiting to use boiled water (cooling time) but they do not indicate what portion of this time is spent with water at a “boil” or if there were differing user definitions of “boil” as indicated in previous studies [19].

There are significant difficulties in monitoring and evaluating the correct and consistent use of HWT practices. Literature has highlighted the systematic differences between self-reported and observed behaviors [20–23]. The “social desirability” or “courtesy” bias, where individuals over report positive behaviors, is well documented [20,22,24]. A 2009 study demonstrated lower rates of confirmed water treatment versus self-reported water treatment for both water boiling and chlorine water treatment techniques [25]. In the 2009 study, confirmed water treatment was based on three criteria: a family self-reporting to treat their drinking water in the past seven days, treating their drinking water at the time of the interview and being able to produce treatment-related products [25]. The research suggested that both self-reported and observed measures of water treatment are important to accurately estimate usage rates.

## Methods

Semi-structured interviews were conducted to elicit the opinions and experiences related to carbon credits for HWT from key informants working in WASH and carbon credit development sectors. Interviews explored experience, knowledge and opinions of carbon credits as applied to HWT, with specific reference to the Gold Standard methodology. The interview guide can be found in the Supplemental Materials. The George Washington University Office of Human Research Institutional Review Board reviewed and approved this study IRB#021461. All interviews were audio recorded and participants consented either written or verbally to have their interview audio recorded. Where written consent was not obtained, because participants were remotely interviewed and unable to scan a signed document, audio recorded consent was obtained prior to recording the interview. The George Washington University Office of Human Research Institutional Review Board approved this consent procedure in the application.

Interview participants were selected based on their experience in carbon credit development and/or household water treatment and safe storage. Participants were asked for referrals to other individuals that they felt could offer valuable insight into the issues addressed. In total 24 individuals were contacted with a recruitment email. Initially 16 individuals were identified as potential participants; the remaining eight experts were identified via snowball sampling referrals. Of the 16 originally contacted five individuals did not respond to either the initial recruitment or any attempts at follow-up contact, these were taken as tacit refusals. Two individuals in the original contact list refused based on either lack of expertise or time constraints, both of these individuals referred the authors to individuals they felt were better suited to the study or had adequate time. Of the eight people identified via snowball sampling four participated in interviews. The other four refused based on lack of expertise and time constraints.

Out of the 24 potential participants, 13 respondents were interviewed over a period of six weeks. Interviews lasted between 25 and 84 minutes, the average length of an interview was 49 minutes. Seven respondents were classified as experts in WASH and seven respondents were classified as experts in carbon credits; one expert self-identified as experienced in both fields. Respondents were grouped according to self-reported area of expertise. The broad categories of expertise sought were individuals with knowledge of WASH and those with knowledge of carbon credits and carbon development projects. The authors acknowledge snowball sampling, the use of a single methodology (Gold Standard), and the limited time frame of this study inherently limits the representativeness of the sample. We do not intend this to be a representative sample of the carbon and WASH experts in the field but rather an initial exploration of key themes for further exploration in a more comprehensive study. Table 2 provides detailed information about respondent characteristics. The interviews were audio-recorded, with participants’ permission; transcribed; and reviewed for patterns, commonalities and differences among respondents.

**Table 2:** Background information on study participants, by area of expertise, type of organization and position.

Area of Expertise*	Number (%)
WASH	7 (46%)
Carbon Credits	7 (54%)
**Type of organization**	
University	5 (38%)
Carbon Credit Program Developer/Implementer	6 (46%)
Monitoring and Evaluation Consultant	1 (7%)
Government	2 (15%)
**Position/Primary Role**	
Researcher	6 (46%)
Program Officer/Program Manager	5 (38%)
Executive Leadership	3 (23%)

*One expert self-identified as both expert in WASH and carbon credit development, and one expert holds positions at both a University and a carbon credit development firm and is therefore included in both counts.

## Results

The interviews focused on key areas of interest in the Gold Standard methodology. Two broad themes that emerged from these interviews relate to what measurements are collected for monitoring the use of HWT as well as verifying the emissions reductions achieved, and the purpose or goals of carbon credit projects. The results highlighted the controversy surrounding monitoring and evaluation procedures in carbon credit projects, see Table 3 for key quotes from study informants. WASH and carbon credit development experts differed in their views of the most appropriate indicators and procedures for monitoring usage rates as well as the BWBT. These two groups demonstrated near consensus in their responses regarding suppressed demand.

**Table 3:** Key quotes from study key informants.

*With all household water treatment I don’t believe anything people report because I think that it’s just like hand washing*, *people have a fairly good idea of what you want to hear*. *There’s a lot of courtesy bias […] so I don’t put a lot of stock in reported answers*.*” WASH Expert #1*
*“There’s definitely what we call courtesy bias in the surveys and I’ve seen that first hand*. *[…*.*] So the observation makes a lot more sense the only kind of problem that I think arises is that it depends on the experience of the person doing the observing*.*” CC Expert #1*
*“A consistent user would be someone who uses it every day*. *[…*.*] I would say a user is someone who has it setup correctly and reports at least some use but a consistent user is every day*. *“WASH Expert #3*
*“I think it’s important to be up front about where the fictions are […] this concept of suppressed demand is fiction but as long as everybody’s ok with it then I don’t see a problem*. *“WASH Expert #4*
*“The most important thing in terms of securing a public health impact for water treatment is consistent use over time*. *[…] So I think it’s a very good public health argument to make for insisting on very high levels of adherence you know to actually achieve health impacts*.*” WASH Expert #4*
*“These are businesses right so these businesses are going to invest money […] if you want to incentivize business to participate in this market and these activities they have to have a pretty reasonable understanding of what the rules are*.*” CC Expert #2*
*“No I don’t think boiling for 10 minutes is something that anybody does anywhere in the world*. *[…] I would be extremely surprised if anybody boiled their water for 10 minutes*. *“WASH Expert #4*
*“The real point of you know water based carbon project is to deliver safe water*.*” CC Expert #3*

### Monitoring and evaluation: Water treatment usage surveys

The water treatment usage survey is designed to assess whether a household is a regular user of the HWT and therefore can be counted in the emissions reductions. The guidelines advise using both observed and self-reported measures to evaluate a household’s usage practices. Five WASH experts (83%) identified the importance of observed measures, citing the unreliability of self-reported measures:

*“With all household water treatment I don’t believe anything people report because I think that it’s just like hand washing, people have a fairly good idea of what you want to hear. There’s a lot of courtesy bias […] so I don’t put a lot of stock in reported answers.”*

*(WASH Expert #1)*

*“I think we know from previous research that reported outcomes are very unreliable. I don’t care if its hand washing with soap or water treatment or use of a latrine or anything. I treat any reported outcome with a grain of salt. I strongly believe the only meaningful outcomes are those that you can observe directly or through different technological tools.”*

*(WASH Expert #2)*

Carbon credit experts generally acknowledged that observed measures were better than self-reported measures and that courtesy bias was an issue in self-reported data. Five of the carbon credit experts identified both observed and self-reported measures as necessary for confirming usage of a technology (71.4%). Three carbon credit experts (42.8%) also pointed to the potential flaws and issues of gaming that could arise for observed measures:

*“There’s definitely what we call courtesy bias in the surveys and I’ve seen that first hand. [….] So the observation makes a lot more sense. The only kind of problem that I think arises is that it depends on the experience of the person doing the observing. [….] What people tend to do is they put it [the filter] in the middle of the room and they put a like a cloth over it […] The verifier took this to mean that the water filter, as an observation, […] not being used.”*

*(CC Expert #1)*

*“Observation I think is very good. It’s just hard to write an observation into specific questions […] you also want to give some leeway to your surveyors to have the option to say ‘do you think they’re users or not.’ In general you pick up on a lot of cues that aren’t easy to write in surveys and the self-report is just as important. [….] I do think people demonstrating [use] is a fairly good measure because you’re able to sort of observe if they go through the process correctly. I think they’re both important to include but I’m not sure you can ever have one without the other.”*

*(CC Expert #3)*

The idea of a familiarity with the local context for observational measures cut across the two disciplines. One WASH (16.6%) and two carbon credit experts (28.5%) described how valid observational measures were predicated on the observer understanding what they were seeing.

*“If you have someone who’s not familiar with it doing the observation you might end up with results that are not representative. I think that the person doing the observing needs to have sufficient familiarity with the local context to be able to do it well.”*

*(CC Expert #1)*

*“Just an example of a problem that has come up in solar water disinfection [….] the people […] knew […] the promoters wanted them to be treating their water with SODIS and so what often families would do would be to leave bottles of water on the roof and they’d just never take them off. They’d just leave them there and so anytime the promoters came by you know they’d be like ‘yeah look we’ve got water and you know we’re treating it’ but it turned out that in a lot of cases they were just leaving it up on the roof and never really drinking it. So you know even these observed measures can be gamed and imperfect.”*

*(WASH Expert #1)*

### Monitoring and evaluation: Defining use

The Gold Standard usage survey guidelines provide example questions to determine HWT usage and also state “the project proponent should decide appropriate frequency of water treatment to be considered as usage, based on local practices and circumstances”[8]. When asked if there should be an absolute definition of usage, responses were mixed and did not follow a distinct pattern based on field of expertise. One WASH expert noted that the availability and volume of safe storage would affect the frequency of technology use in some cases:

*“Say something made 50 liters at a time of safe water and then there was a safe storage container that couldn’t be contaminated and the households might use that for two or three days […], I can see how you could argue that usage would be every other day or every three days. I think right now with the products on the market […] the vast majority of the filters don’t produce enough water to last more than one day, except the biosand filters. [….] Maybe the better way to say this is there should be treated water available in the household every day.”*

*(WASH Expert #3)*

Another key informant was more emphatic in recommending an absolute definition of use.

*“Yes […] what we’re doing is we want to take public resources to pay for interventions that have a certain outcome and I think we owe it to the donors to give them an accurate representation of what they’re paying for. And so I think there should be stringent guidelines for doing it.”*

*(WASH Expert #2)*

Responses from carbon credit experts were mixed on whether an absolute definition was appropriate but six of the carbon credit experts (86%) identified the use of a cap or upper limit as an important tool in defining an appropriate usage rate:

*“I think that there’s an upper limit that you would tentatively give credit for, I mean if people typically use 10 liters a day of which 1.5 or 2 is for drinking and 8 is for hygiene and cooking and whatever else. [….] I think providing a larger number of credits is not necessarily in anyone’s interest.”*

*(CC Expert #1)*

*“It is somewhat context specific and you see people using different amounts of water during different seasons so it’s kind of hard to apply a rule but I think you could say something like you know the WHO says 6 liters per person per day is reasonable […].”*

*(CC Expert #3)*

Two of the six carbon credit experts interviewed noted how important the usage rate is to the financial viability of a carbon credit project:

*“Now if somebody is an inconsistent user does that make that filter inappropriate to earn carbon credits? I don’t think so. If somebody is using it half as often as what they should be doing for consistent use that doesn’t mean that that should invalidate those carbon credits. It just means that [the] implementer should work harder to get use up. [….] I think that that would be too much of a stick against an implementer who is trying really hard to get usage numbers up [.…] These are businesses right so these businesses are going to invest money […] if you want to incentivize business to participate in this market and these activities they have to have a pretty reasonable understanding of what the rules are.”*

*(CC Expert #2)*

*“I think people still can certainly be considered users if they’re not practicing perfect safe storage practices and that’s kind of an ongoing public health challenge that we try to address. I think […] imposing really strict criteria on that would be pretty difficult to make the carbon project viable.”*

*(CC Expert #3)*

### Monitoring and evaluation: Defining consistent use

Another topic covered in the usage survey guidelines is the rate of usage. This is intended to “rule out users who report low frequency of usage of the project HWT unit.” When asked how frequently a user or household should be employing a technology to be considered a regular user there were marked differences in the responses of carbon credit and WASH experts. WASH experts were more likely to give concrete time frames of when the technology was last used while carbon credit experts gave more nuanced responses. Two WASH experts (33.3%) described consistent use as every day:

*“A consistent user would be someone who uses it every day. [….] I would say a user is someone who has it set up correctly and reports at least some use but a consistent user is every day.”*

*(WASH Expert #3)*

*“I think every day. [….] All the benefits go to those who are regular users. You know just intermittent use doesn’t cut it because you’re going to get exposed to pathogens if you aren’t using these regularly.”*

*(WASH Expert #2)*

One carbon credit expert also felt that daily use was a reasonable measure of regular use but they contextualized their response in terms of availability of a safe storage container:

*“Well why are you filtering the water? It’s to avoid getting sick right. And […] you should be drinking water every day […] I suppose if you had adequate safe storage then you wouldn’t have to use [the filter] every day but the vast majority of people […] doesn’t have adequate safe storage for more than a day […] maybe two days […] I think daily is a reasonable standard.”*

*(CC Expert #1)*

Three carbon credit experts gave examples of how consistent use is difficult to measure and would require contextualization. These responses indicated that a certain volume of water provided might be a better measure of consistent use:

*“We’ve got plenty of households where it’s just one young man living there […] he may well be a consistent user using it all the time and he only fills it up once every third day he may well only drink 2 liters of water a day that’s totally practical and reasonable whereas a family of 10 might use it every day and still not be using it enough to be consistent users. So there’s some minimum amount of water delivered per adult per day that can be measured as a consistent user.”*

*(CC Expert #2)*

One-third of the WASH experts framed consistent use as a measurement of the volume of treated water consumed rather than as a frequency of technology use. These experts also noted consistent use was important to measuring the health impacts of an intervention:

*“The most important thing in terms of securing a public health impact for water treatment is consistent use over time. [….] There’s probably very little difference in risk between somebody who uses a water treatment device 50% of the time and 0% of the time. You’re not going see a big difference in risk there, you will see a difference in risk when you go from 100% to 90% but if you get much below 90% the difference between that and 0 is very limited. So I think it’s a very good public health argument to make for insisting on very high levels of adherence […] to actually achieve health impacts.”*

*(WASH Expert #4)*

### Monitoring and evaluation: Baseline water boiling test

The Gold Standard methodology defines the baseline water boiling test as the “quantity of fuel required to purify by boiling one liter of water for 10 minutes using technologies and fuels representative of the baseline scenario”[7]. There was almost universal acknowledgement that 10 minutes was an overestimation of the time it would take to make water microbiologically safe to drink. The United States Centers for Disease Control and Prevention (CDC) guidance for boiling water is to bring the water to a boil (bubbles come from the bottom of the pan to the top) and then keep at a rolling boil for one minute before storing safely [26].

Five WASH experts (83%) noted that while 10 minutes would make the water safe, this would overestimate the amount of time required to purify water:

*“No, one minute is more than adequate. You know really once water gets up to the boiling point you have killed virtually everything in the water. […] Ten minutes is overkill […] you’ll overestimate the benefit you’re going to get.”*

*(WASH Expert #2)*

*“I think you don’t need to boil for 10 minutes for microbiological safety. […] I mean you don’t even really have to reach boiling temperature unless you’re at super super high elevation […] but most of the time you don’t even need to reach full boiling to have microbiological safety you just need to reach pretty high up but they tell you to boil because that’s a visual indicator so I would say the ten minutes that’s a pretty conservative number.”*

*(WASH Expert #3)*

Six carbon credit experts (83%) also acknowledged that 10 minutes is not reflective of the time it takes to purify water. One carbon credit expert (14.3%) referenced the business opportunity that carbon credits present and the importance of this measurement for that consideration:

*“It is high. It doesn’t take 10 minutes to disinfect water but again if you were to say it should be a minute you just deleted 90% of the carbon credits and probably made the project not financially viable. It would certainly cut into the potential for this. This is not a high-margin activity. Nobody’s getting rich off of this.”*

*(CC Expert #2)*

### Monitoring and evaluation: User boiling practices

When asked if 10 minutes of boiling was reflective of user practices in the field, five WASH experts (83%) rejected 10 minutes as being an accurate estimate of user practices. These experts agreed that 10 minutes was an overestimation of how long users in the field boil water.

*“No. No one boils their water for 10 minutes.”*

*(WASH Expert #3)*

*“In Africa almost nobody treats their water by boiling. They’ll say they do and sometimes they may do it but it’s a very uncommon practice. […] And you know if they do boil it, I doubt that they boil it for 10 minutes.”*

*(WASH Expert #2)*

*“No I don’t think boiling for 10 minutes is something that anybody does anywhere in the world. […] I would be extremely surprised if anybody boiled their water for 10 minutes.”*

*(WASH Expert #4)*

Carbon credit experts acknowledged that 10 minutes was likely too high to reflect user practices but they also frequently mentioned experiences conducting field observations and the complexities around people measuring and understanding their own boiling practices:

*“Depends on how much wood they’ve got, how much time they’ve got, how much water they’ve got, do they want to drink it now or later? [….] it varies a lot but other people would leave it there boiling for a while because they were away or something so it’s pretty variable. But I’d say if anything, common practice would be less than 5 minutes rolling boil rather than more.”*

*(CC Expert #1)*

### Goal of carbon credits: Suppressed demand

Suppressed demand is a policy tool that allows for carbon credit developers to base claimed emissions reductions on a theoretical scenario rather than a baseline grounded in evidentiary practice. The idea is that current boiling practice is limited by availability and/or affordability of fuel, and if fuel were available, people would boil their water. So providing an alternative to boiling can be counted as carbon credits even when people are not currently boiling. In other words the credits are issued for emissions avoided rather than emissions reduced. While this practice has received criticism is should be noted that the shortcomings have been acknowledged by members of the scientific community [27]. The policy objectives that suppressed demand is intended to achieve are fundamental goals of the Kyoto Protocol, the idea that least developed countries must be given special consideration and assistance in order to effectively and fully participate in the carbon market [27]. Proponents acknowledge that use of suppressed demand overstates the actual reduction in greenhouse gases but that enabling the participation of those households traditionally overlooked by market based interventions in least developed countries is an essential enabling factor and a key justification for the use of suppressed demand [15, 27]. In acknowledging the shortcomings and general misunderstanding of this policy, some have called for the incorporation of more rigorous evaluation tools as well as the use of a higher standard of water quality measurement [27].

Two WASH (33%) experts acknowledged the value and the theoretical basis of suppressed demand. These experts expressed that due to the complexity of HWT, applying suppressed demand to water treatment projects is difficult:

*“I think that it’s a theoretical concept. […] I think it would be easier to justify if there were some sort of empirical evidence that […] this happens in populations […]. But it seems to me like a sort of a theoretical proposition that doesn’t have a lot of evidence behind it. […] Another example like […] Guatemala, […] that country’s got a huge range of wealth in it. There are some of the poorest people in the world and some of the wealthiest people in the world. Well the wealthier people don’t boil their water and even the people in the middle don’t boil their water. They get water delivered to them [….] so I’m not sure that I buy that the sort of step above abject poverty is not necessarily boiling their water. [….] I think that this is kind of pie in the sky a little bit theoretical.”*

*(WASH Expert #1)*

*“It makes a lot more sense to me to apply [carbon credits] to less use of carbon in general which is what gets you to these improved cook stoves [….] treating water to me is taking kind of a circuitous route, particularly given the complexity of water treatment practices.”*

*(WASH Expert #2)*

All of the carbon credit experts and four WASH experts (66%) focused on the use of carbon credits as a morally and ethically justifiable tool for development. While they acknowledged that the concept and the measurements were not perfect, the benefits and the obligation to fulfill development goals from a human rights perspective appeared to outweigh these concerns:

*“You know suppressed demand as a concept kind of stretches the limits of plausibility with household water treatment which given what we know about what people actually do. […] I think many of us in the water sector would prefer to say look […] lets approach this problem honestly and […] put in water projects because putting in water projects is the right thing to do and it impacts health. There’s […] skepticism and frustration that we need to employ this fiction of suppressed demand in order to make carbon credits work for water. I think people resent that because it is based on […] this fiction essentially that people are boiling a whole lot of water using non-renewable wood resources which we know they’re not doing so […] I see all of this as a vehicle for doing the right thing that is based on assumptions that are not realistic […] I think it’s important to be upfront about where the fictions are […] this concept of suppressed demand is fiction but as long as everybody’s ok with it then I don’t see a problem.”*

*(WASH Expert #4)*

*“It’s definitely important […] you know the real point of water based carbon projects is to deliver safe water. […] If you think about it in a more simple context outside of water […] you don’t want to encourage the behavior of countries that are on a development pathway where it’s cheaper to go dirty first and then they see even more incentive because they can go from a high baseline of pollution and claim carbon credits. […] I think it’s just most important in thinking about in the context of making financing accessible and enabling a creative way of financing safe water and it’s a reasonable approach but it’ll never be exact.”*

*(CC Expert #3)*

### Goal of carbon credits: Water quality

Experts were asked whether water quality is an important consideration in carbon for water projects. This question yielded interesting results regarding experts’ understandings and beliefs about the ultimate goal or purpose of carbon for water projects.

Carbon credit experts were united in their view that measuring water quality is essential in these projects because the ultimate goal of the project is to bring clean, safe water to under resourced areas:

*“Most important is that we try to provide the highest quality of water so our view is that the highly protective WHO rating is the appropriate metric for the intervention. [….] What’s the point of these fancy water filters? Fundamentally it’s not about carbon credits. Fundamentally it’s not about having an appliance in your home. Fundamentally it is about health benefits and the absence of disease associated with clean water.”*

*(CC Expert #2)*

*“It’s definitely important […] the real point of […] water based carbon project is to deliver safe water.”*

*(CC Expert #3)*

*“I definitely think that’s [water quality] important and if we’re going to put together […] elaborate crediting mechanisms to enable safe water, it better be safe. If it’s not safe you’re defeating the whole purpose.”*

*(CC Expert #4)*

Two WASH experts also expressed the importance of water quality measures and the relationship to reduction of disease. However, they were more likely to talk about this as a secondary or additional objective of a carbon credit project.

*“I mean it doesn’t seem to me to relate directly to the amount of wood they consume or biomass they consume. […] I think that a lot of the programs that advocate for these household water treatment technologies do it on the basis of health […] so if that’s another stated goal of their project, but I don’t think that that would be central to the actual carbon credit audit.”*

*(WASH Expert #1)*

*“I do think so. I do because […]if your policy objective is to have less black soot in the air that’s one thing […] but if the reason for doing carbon credits is to have an additional policy objective, which is reduction of diarrheal diseases particularly in young children, then the water quality can be a proxy for that. […] I think there are two policy objectives here and I think having a measure of water quality kind of holds people’s feet to the fire whether they’re meeting the program objectives.”*

*(WASH Expert #2)*

## Discussion

Utilizing carbon credits to finance safe drinking water technology has been controversial since its inception. Some WASH experts, as well as independent NGOs and other development proponents, have criticized the methodologies used to calculate emissions reductions achieved from these projects. By interviewing carbon credit experts and WASH experts, we were able to gain a clearer picture of the areas of disagreement on how these projects should proceed.

The most marked difference between these two groups was in their approach to the business model of carbon credits. Half of the carbon credit experts discussed considerations related to the financial viability of these HWT projects. These carbon credit experts expressed the need to incentivize developers to participate in the market for these projects. On the other hand WASH experts never discussed the financial or business related aspects of these projects. This is an interesting dichotomy given the need to balance both technical aspects of these projects, such as water quality monitoring and consistent usage, with long-term financial sustainability. WASH experts were united in their view that observed measures of usage were superior in rigor and validity to self-reported measures. This is consistent with current literature on self-reported measurements for hand-washing and hygiene behaviors [20,22,24]. In contrast, carbon credit experts acknowledged the short-comings of self-reported measures; however, based on their experience applying these criteria in the field, they felt both observed and self-reported measures were important to context and accurate representation of a given field situation because observed measures could also be gamed or misinterpreted.

Recent studies have explored the use of remote sensing technology to reduce bias and improve monitoring data for interventions requiring confirmation of self-reported behaviors [23,28]. The newest application of this technology is specifically for cookstove and water filtration interventions like those employed in projects registered for carbon credits [28]. The first published reports of field tests indicate that reported use continues to overestimate the usage rate objectively confirmed by the sensors. This technology could not only serve as an independent verification of self-reported data but also could reduce the possibility of gaming in observed measures.

Carbon credit experts in our sample were more likely to be based at private or non-profit organizations that are primarily focused on program development and implementation (86%) compared to WASH experts (33%), who were more likely to be from Universities or research based institutions. It could be that the carbon credit experts interviewed have more experience with the application of methodologies and monitoring in real-world scenarios. Alternatively, the incentives to have a successful program may have biased the carbon credit experts towards being more willing to accept households as users. Carbon credit experts’ responses indicated that in general they approach carbon credit projects as a market-based mechanism, intended to make a profit or at the very least break-even. This business model approach was reflected in carbon credit experts’ responses to questions regarding usage rate calculations and the baseline water-boiling test.

Both carbon credit and WASH experts acknowledged that 10 minutes was not reflective of user boiling practices in a field setting. While there is agreement that 10 minutes is an overstatement, there is again a fundamental difference in approach and goals of these two groups. While carbon credit experts acknowledged the shortcomings of a 10 minute parameter they often contextualized this based on field experience in collecting these measurements. Current literature is inconclusive regarding both a standard user definition of boiling as well as clear references for standard user boiling practices [17–19]. Until there is a clearer picture both globally and regionally of user boiling rates and practices, this measurement will likely remain controversial.

All carbon credit experts interviewed maintained that while the carbon credits generate income, the ultimate goal is provide clean water and improve health. When asked whether water quality should be a consideration in carbon credit projects, all carbon credit experts believed water quality was not only important but also an illustration of the fundamental goals of the project.

Two WASH experts (33%) acknowledged that water quality was a fundamental necessity for improving health, but it was not viewed as the sole purpose of carbon for water projects. These WASH experts agreed that monitoring water quality is important in holding these projects accountable but they also frequently mentioned that the environmental objective of carbon credit projects is to reduce emissions and that more effort should be placed on understanding changes in emissions as a result of carbon credit projects. The remaining five WASH experts agreed that monitoring water quality was important in any HWT provision project. They expressed that measuring and monitoring water quality was important to providing safe, clean drinking water but they didn’t address this in terms of the goals of the carbon credit project. As noted by Yeo (2013), there is the potential that health benefits of carbon for water projects are overstated and similarly that water quality is only part of the equation for reducing disease and improving health. Other factors—hand washing and hygienic sanitation—are also important factors for improving WASH-related health outcomes [28].

While the use of suppressed demand has been controversial [30] there was agreement that though it is considered “fiction” in terms of baseline measurement, the moral and ethical necessity of bringing safe water to underserved populations justifies the mechanism. While this view illustrates a consensus between the two groups, this contradicts the goal of carbon credits that aims to reduce emissions. Previous work points to three main concerns with the use of suppressed demand: 1) the need to prove that emissions would have resulted without project implementation 2) the use of suppressed demand in populations where boiling is not the predominant means of water purification in the baseline scenario and 3) the assumption that suppressed demand contributes to a “leapfrog” in the development pathway. “Leapfrogging” implies that users of the HWT who were not previously boiling water, are skipping this step because they would be boiling if they had access to the resources (monetary and time) to boil water [29]. Given the importance of greenhouse gas emissions, this justification deserves additional attention and debate.

There were several limitations to this research. The relatively small sample size of the study limits its generalizability. By focusing our questions on a single methodology, Gold Standard, it is possible that experts were missed who could have offered a valuable, different perspective on carbon credits and WASH more generally. In drawing our sample we utilized professional networks and referrals from respondents. This snowball sampling method may have systematically excluded important individuals from the data collection process. HWT technologies vary in many respects and many of the respondents qualified their statements saying certain factors and considerations would vary given any specific treatment technology. This was intended to be a general exploration of the application of carbon credits to HWT. It could prove valuable in future studies to explore specific treatment technologies in greater depth. This could potentially yield a more diverse sample of experts as individuals engaged in WASH research may be more or less familiar with any given treatment technology.

The results of this study suggest that while WASH and carbon credit experts’ approaches to and understanding of carbon credits differ, there are areas of commonality. It is a natural tendency for disciplines and communities of practice to become narrowly focused in their field of expertise, which can provide in-depth understanding, but may also inadequately capture the complexity. While there was clear agreement that carbon credits are a valuable tool for providing accessible financing to the world’s poorest, the results highlight that more efforts are needed to bring the WASH and carbon credit sectors together to develop more agreement in the methods applied in carbon credit projects for drinking water quality.

Based on this research with sector experts in WASH and carbon credits, we recommend that more effort be placed on building consensus on the underlying assumptions for obtaining carbon credits from HWT projects, as well as the methods for monitoring correct and consistent use of the HWT technologies to support public health impact.

## Supporting Information

S1 Text Title Interview Guide. (DOCX)
