# Supplementary material for: CO2 and H2O: Understanding Different Stakeholder Perspectives on the Use of Carbon Credits to Finance Household Water Treatment Projects
Source: PLoS One. 2015 Apr 30;10(4):e0122894. doi: 10.1371/journal.pone.0122894 (PMC4416006; doi:10.1371/journal.pone.0122894)
Supplement: S1 Text Title — (DOCX) [file pone.0122894.s001.docx]

Thank you for participating in this study. The study aims to assess the monitoring and evaluation practices of carbon credit methodologies as applied to household water treatment technologies. I would like to record this interview so that I can correctly capture your answers.

1. Do you have experience or knowledge of carbon credits as a tool for development?
   1. Please provide a brief description of your knowledge or experience with carbon credits as it applies to household water treatment projects
2. Are you familiar with The Gold Standard certification for carbon mitigation projects?
   1. If so, please provide a brief description of your experience or knowledge of The Gold Standard as it applies to household water treatment projects.

The next set of questions is based on information contained in the Gold Standard Methodology. Each question describes either a portion of the methodology or a scenario in which the methodology is applied. Please read each question carefully and respond to the best of your ability based on your expert opinion and your experience in the field.

**Background information**: The Gold Standard recently released updated guidelines for usage surveys in household water filtration projects. I would like to ask you a few questions related to these new guidelines. Additionally the Gold Standard methodology underwent revision between 2010 and 2011. An updated version of the methodology was issued in November 2011. I will ask you questions related to the 2011 version of the methodology. I have provided portions of the 2011 methodology below in boxes; please refer to these boxes when necessary. Some sections also refer to a portion of the background information I sent you. I have noted which background document corresponds to each section. I’m trying to gain an understanding based on your opinion and experience and I understand that some of this material may or may not be familiar to you.

**Section I. Usage Survey Questions**

**Document: Gold Standard Guidelines on Carrying out Water Usage Survey**

1. What indicator(s) do you think should be employed to classify households as users of a filtration technology?
2. The guidelines state “the Project Proponent should decide appropriate frequency of water treatment to be considered as usage, based on local practices and circumstances.” Based on your experience should there be an absolute definition of usage?
3. How frequently do you think a household needs to use a treatment technology to be considered a “regular user” of this technology?

**Section II. Baseline Water Boil Test**

**Document: Gold Standard Excerpt Box 1 (reproduced below)**

| **Box 1: Baseline Water Boiling Test**  The baseline water-boiling test (BWBT) is conducted to calculate the quantity of fuel required to purify by boiling one litre of water for 10 minutes using technologies and fuels representative of the baseline scenario.  If the monitoring surveys reveal that the same water boiling technologies are prevalent in the baseline and project scenarios, W_b,y_ and W_p,y_ are equal. The BWBT should be updated if monitoring surveys show that water boiling technologies change over time. |
| --- |

**Based on the information contained in Box 1:**

1. Have you ever conducted a baseline water-boiling test? Can you describe this procedure?
2. In your opinion is the parameter of a 10-minute boiling time a valid indicator of microbiologic safety of water? Why or why not?
3. In your experience is 10 minutes an accurate estimate of the amount of time users actually boil water? Why or why not?

**Section III. Quantity of Water**

**Document: Gold Standard Excerpt Box 2 (reproduced below)**

**Based on the information in Box 2:**

The intent of these measurements is to capture how much water regular technology users are consuming. This estimate is essential to calculating emissions reductions as the amount of water that was previously boiled and is now filtered affects the amount of fuel consumed and therefore emissions produced.

| **Box 2 Water Consumption Field Test**  The water consumption field test (WCFT) is similar to the Field Test, except project-supplied clean water consumption volumes and boiling is measured rather than fuel consumption. The WCFT is conducted with end users representative of the project scenario target population and currently using the project technology. Three different volumetric variables are measured, as indicated by the equations below:  **Q_p,y_**  Quantity (Q) of safe water in litres consumed in the project scenario (p) and supplied by project technology per person per day (y)  **Q_p,rawboil,y_**  Quantity of raw or unsafe water boiled (Q) in the project scenario (p) per person per day (y)  **Q_p,cleanboil,y_**  Quantity of safe (treated, or from safe supply) water (Q) boiled in the project scenario(p) per person per day (y) |
| --- |

Have you ever conducted a water consumption field test?

**Field Test Measured Usage**

1. How would you conduct a water consumption field test (WCFT) (what would be the procedure for conducting this test)?
2. Would this replace reported usage?
3. Would the method for conducting a WCFT differ based on the filtration technology?

**Section IV. WHO Toolkit**

**Document: WHO Toolkit, and Gold Standard Requirements for Treated Water Quality**

1. Are you familiar with the WHO toolkit for monitoring household water treatment and safe storage programs? (If yes please briefly describe how you use the toolkit?)

2. Do you think this is a useful tool?

3. Do you have any recommendations on improving the WHO toolkit?

4.The current Gold Standard usage survey guidelines require monitoring of household water storage practices (page 3 usage guidelines). In your opinion is this an important consideration?

5. A recent update to Gold Standard guidelines requires monitoring and evaluation of water quality. In your opinion should water quality be a consideration in carbon credit projects?

6.The guidelines suggest that host country standards should be a first point of reference in measuring water quality. In your experience are host country standards, if in existence, significantly different than WHO guidelines?

7. In your opinion what are the most important measures of water quality?

8. How often should water quality be measured?

**Section V.**

**Document: Gold Standard Excerpt Box 3 (reproduced below)**

| **Box 3: Suppressed Demand**  In many developing countries the level of energy service is not sufficient to meet human development needs due to lack of financial means and/or access to modern energy infrastructure or resources. This concept is known as “suppressed demand” and is described in Annex 2 of the methodology. The methodology allows for a baseline scenario to be assessed in terms of suppressed demand, if evidence is provided that the project technology users, or a “cluster” of such users within the project population, are otherwise deprived of a reasonable level of human development (or an acceptable benchmark) in comparison to their peers, and provided that there is likelihood of avoided future emissions, as for example if the standard of living is increasing for some of the project population or their peers outside of the project boundary. In such case, the level of thermal energy consumption in the baseline scenario will not correspond to the preproject situation but to a satisfied demand level, which will be equal to or lower than the project level of satisfied demand.  Author’s Example: As part of an emissions reduction project the household receives an improved cookstove. The improved cookstove requires less fuel than the baseline technology. The cost of the fuel required to boil water on the unimproved stove limited the amount of water the household was able to boil. Prior to receiving the improved cookstove the household boiled the equivalent of 2 liters of water per person per day. The improved cookstoves uses less fuel for cooking and cooks faster than the original stove, save the household time and money. After receiving the improved cookstove the household boils 6 liters of water per person per day. The household consumes more fuel in total every week using the improved cookstove than the original stove because they can now afford increased amounts of wood based on monetary and time savings from the modified stove. Thus while the overall level of consumption and emissions has risen the household’s standard of living has increased to a satisfactory level. |
| --- |

1. Are you familiar with the concept of suppressed demand?

2. If yes: Are you familiar with this tool as applied to water treatment technologies?

3. Do you feel the concept of suppressed demand is valid when applied to household water treatment projects? Why or why not?
